# Supplementary material for: A Velocity Stretch Reflex Threshold Based on Muscle–Tendon Unit Peak Acceleration to Detect Possible Occurrences of Spasticity during Gait in Children with Cerebral Palsy
Source: Sensors (Basel). 2023 Dec 20;24(1):41. doi: 10.3390/s24010041 (PMC10780611; doi:10.3390/s24010041)
Supplement: Supplementary file 1 [file sensors-24-00041-s001.zip › sensors-2725530-supplementary.pdf]

Figure S1: Superposition of stretches of one subject. Procedure of T_{vmt} determination based on EMG (blue lines) or on kinematics (green lines) for the 3 stretches of one subject's trial. For ease of reading, only one EMG-Onset method is presented. First row: EMG recordings of the Soleus muscle. Muscle activity is in red, while the blue dashed lines represent the EMG-Onset minus 30ms. Second row: Musculo-tendon acceleration (a_{mt}) of the Soleus muscle. The green dashed lines represent the maximum. Third row: Second derivative of the passive moment. The black dashed lines represent the maximum, which we consider to be the catch. Fourth row: Musculo-tendon velocity (v_{mt}) of the Soleus (*Sol*) muscle. The green and blue dashed or dash-dotted lines represent the musculo-tendon velocity thresholds (T_{vmt}) determined through MaxAcc and EMG-Onset methods respectively. T0: beginning of the stretch. Stretch 1: solid line. Stretch 2: dashed line. Stretch 3: dotted line.

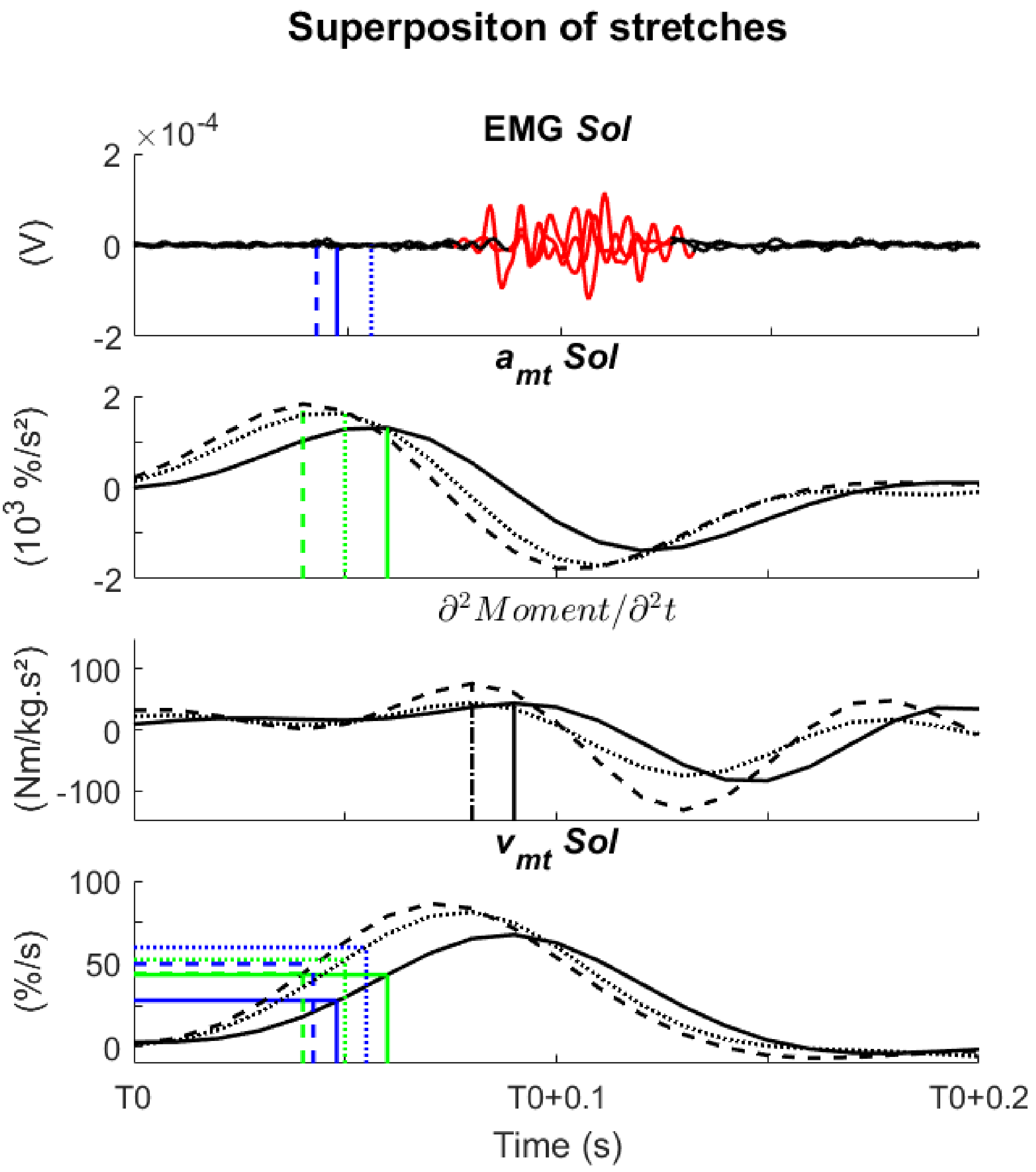

Figure S2: Comparative boxplots of studied parameters between the three methods for all the positions. Following figures present the T_{vmt} values (in %/s), the number of T_{vmt} exceedances per gait cycle (in /gait cycle) and the proportion of the gait trial time (in %) where v_{mt} is exceeding T_{vmt} for the different positions.

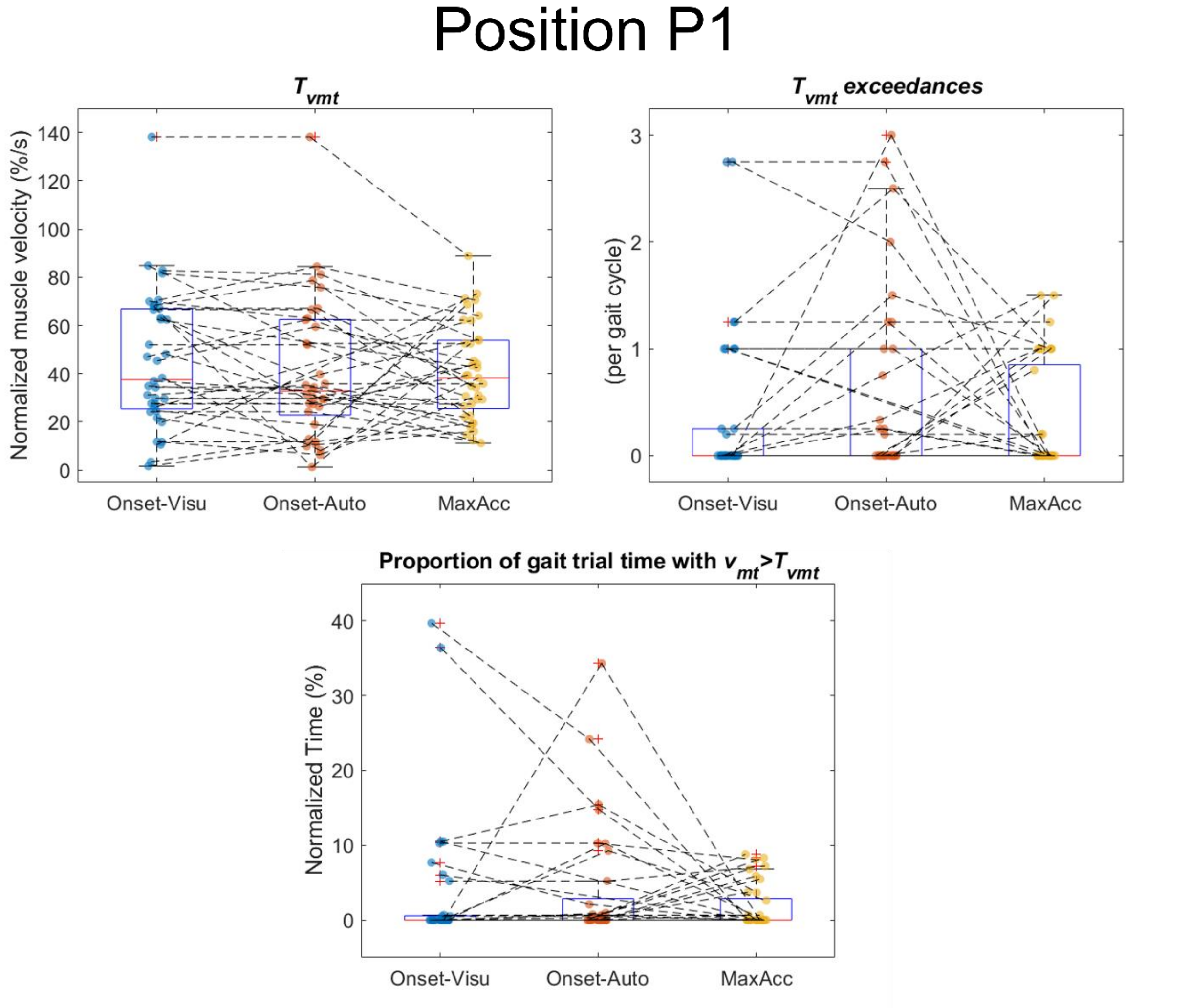

Position P2

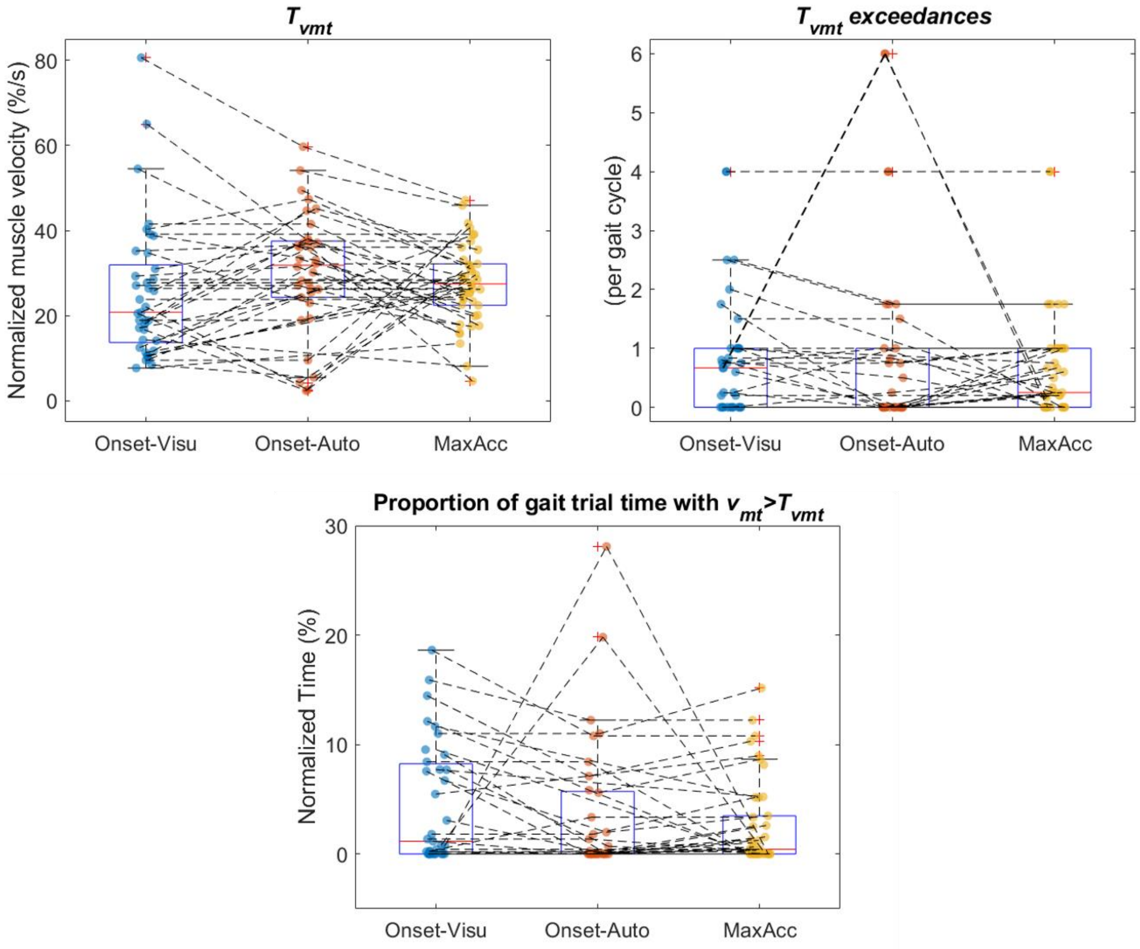

T_{vmt}
Normalized muscle velocity (%/s)
0
20
40
60
80
Onset-Visu
Onset-Auto
MaxAcc
T_{vmt} exceedances
(per gait cycle)
0
1
2
3
4
5
6
Onset-Visu
Onset-Auto
MaxAcc
Proportion of gait trial time with $v_{mt}>T_{vmt}$
Normalized Time (%)
0
10
20
30
Onset-Visu
Onset-Auto
MaxAcc

Position P3

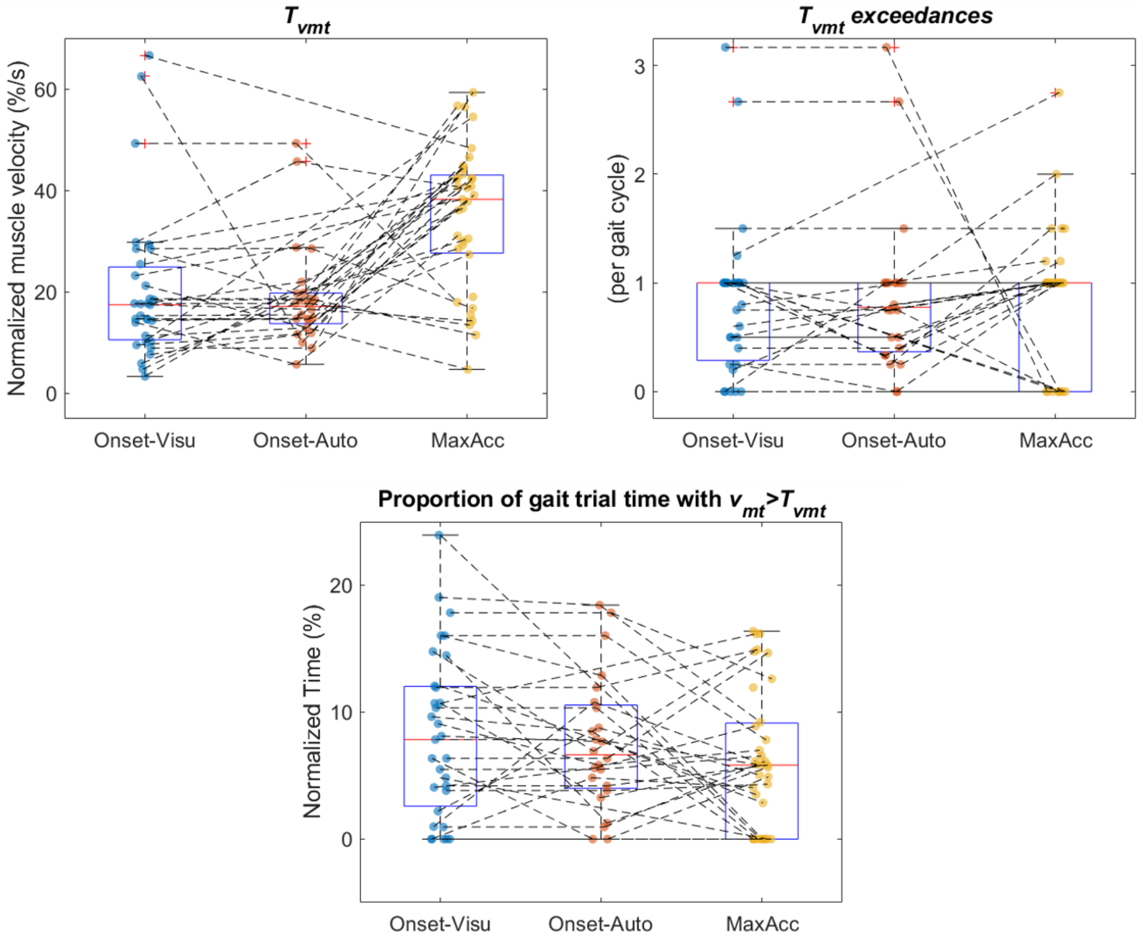

T_{vmt}
Normalized muscle velocity (%/s)
0
20
40
60
Onset-Visu
Onset-Auto
MaxAcc
T_{vmt} exceedances
(per gait cycle)
0
1
2
3
Onset-Visu
Onset-Auto
MaxAcc
Proportion of gait trial time with $v_{mt}>T_{vmt}$
Normalized Time (%)
0
10
20
Onset-Visu
Onset-Auto
MaxAcc

Position P4

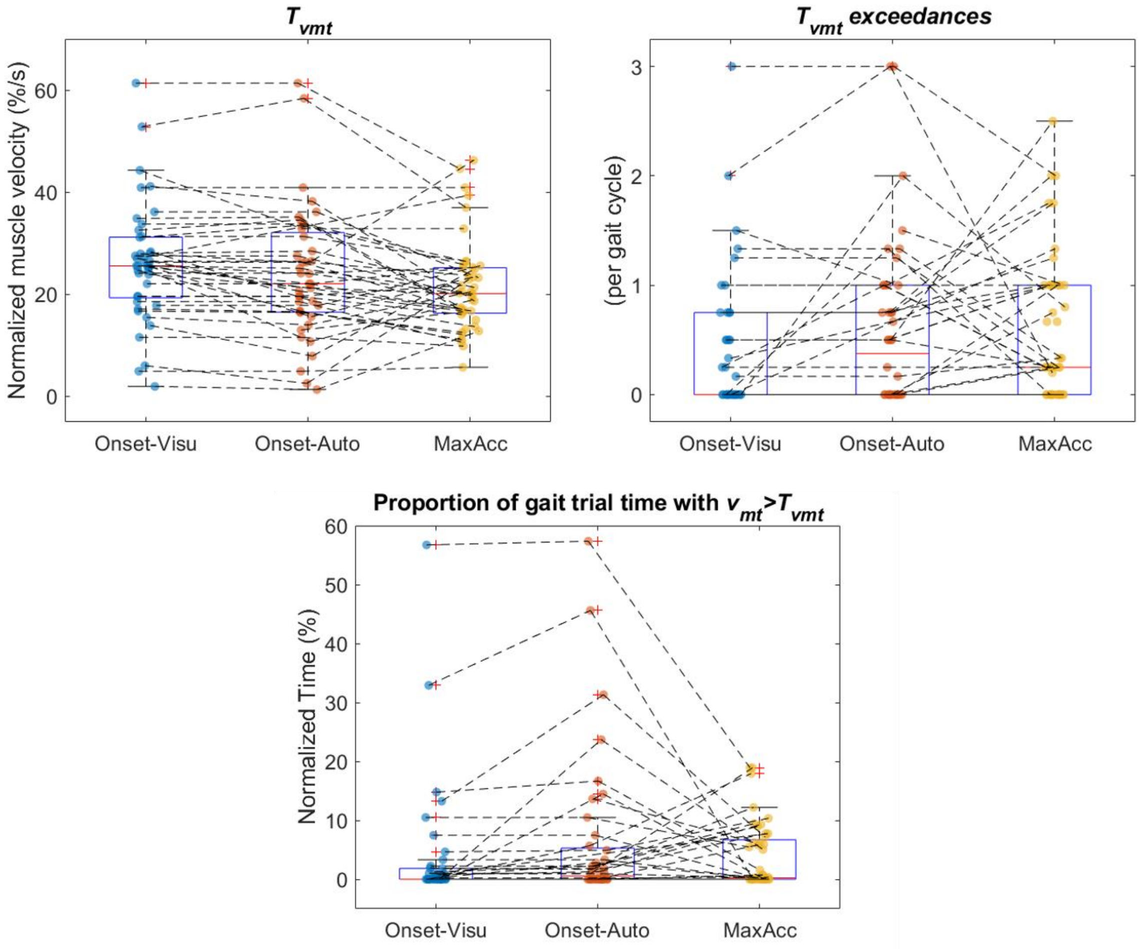

T_{vmt}
Normalized muscle velocity (%/s)
0
20
40
60
Onset-Visu
Onset-Auto
MaxAcc
T_{vmt} exceedances
(per gait cycle)
0
1
2
3
Onset-Visu
Onset-Auto
MaxAcc
Proportion of gait trial time with $v_{mt}>T_{vmt}$
Normalized Time (%)
0
10
20
30
40
50
60
Onset-Visu
Onset-Auto
MaxAcc

Table S1: Pre-T0 detailed values. Following tables present the delay (in ms) between the timing of beginning of the stretch and the EMG-Onset minus 30ms or maximum a_{mt} values for the different positions. For EMG-Onset-Auto, N/A represents the cases where no EMG-Onset was found. Red cases represent pre-T0 physiological inconsistencies.

Position 1 (n=37)			
**Subject**	**Onset-Visu**	**Onset-Auto**	**MaxAcc**
S1 – Stretch 1	28.0	60.5	100.0
S1 – Stretch 2	27.5	57.0	110.0
S2 – Stretch 1	98.0	91.0	60.0
S2 – Stretch 2	181.5	192.5	160.0
S2 – Stretch 3	42.0	42.0	30.0
S3 – Stretch 1	77.0	76.5	40.0
S3 – Stretch 2	121.0	87.0	50.0
S5 – Stretch 1	105.0	97.5	80.0
S5 – Stretch 2	152.0	152.0	90.0
S5 – Stretch 3	117.0	105.0	70.0
S6 – Stretch 1	32.0	19.5	10.0
S6 – Stretch 2	107.0	90.0	80.0
S8 – Stretch 1	193.0	192.0	120.0
S8 – Stretch 2	106.5	108.0	70.0
S9 – Stretch 1	50.5	-10.5	30.0
S9 – Stretch 2	73.0	48.0	40.0
S9 – Stretch 3	53.5	56.5	40.0
S10 – Stretch 1	61.0	38.0	40.0
S10 – Stretch 2	75.0	82.5	20.0
S10 – Stretch 3	93.5	66.5	40.0
S11 – Stretch 1	162.0	145.0	50.0
S11 – Stretch 2	172.5	174.0	30.0
S12 – Stretch 1	49.5	38.0	60.0
S12 – Stretch 2	199.0	96.0	100.0
S13 – Stretch 1	112.0	109.0	30.0
S13 – Stretch 2	124.0	86.0	50.0
S14 – Stretch 1	108.5	123.5	100.0
S14 – Stretch 2	67.0	67.5	30.0
S14 – Stretch 3	149.0	155.0	150.0
S16 – Stretch 1	51.5	17.5	40.0
S16 – Stretch 2	60.0	60.0	60.0
S17 – Stretch 1	109.5	108.0	110.0
S17 – Stretch 2	55.0	40.0	40.0
S17 – Stretch 3	117.0	58.0	100.0
S18 – Stretch 1	49.5	71.5	70.0
S18 – Stretch 2	38.5	-112.0	60.0
S18 – Stretch 3	43.5	-10.5	70.0

**Median**	**93.5**	**76.5**	**60.0**
**25th percentile**	**51.5**	**48.0**	**40.0**
**75th percentile**	**117.0**	**108.0**	**90.0**

Position 2 (n=42)			
**Subject**	**Onset-Visu**	**Onset-Auto**	**MaxAcc**
S1 – Stretch 1	-14.0	35.5	20.0
S1 – Stretch 2	-21.0	-193.5	40.0
S2 – Stretch 1	69.0	92.5	80.0
S2 – Stretch 2	43.0	34.0	60.0
S2 – Stretch 3	22.0	26.5	30.0
S3 – Stretch 1	84.0	106.5	40.0
S3 – Stretch 2	106.5	70.5	50.0
S4 – Stretch 1	178.0	188.5	140.0
S4 – Stretch 2	110.5	95.0	40.0
S4 – Stretch 3	111.0	85.5	60.0
S5 – Stretch 1	54.5	92.0	60.0
S5 – Stretch 2	98.0	-268.0	90.0
S5 – Stretch 3	153.0	317.0	140.0
S6 – Stretch 1	81.0	81.0	70.0
S6 – Stretch 2	70.0	72.5	60.0
S6 – Stretch 3	74.5	92.0	30.0
S8 – Stretch 1	108.0	139.0	10.0
S8 – Stretch 2	66.5	115.5	10.0
S8 – Stretch 3	127.5	429.0	140.0
S9 – Stretch 1	97.0	49.5	100.0
S9 – Stretch 2	178.0	175.0	180.0
S9 – Stretch 3	94.0	57.0	90.0
S11 – Stretch 1	204.5	133.0	100.0
S11 – Stretch 2	244.0	166.0	150.0
S12 – Stretch 1	73.0	76.0	70.0
S12 – Stretch 2	60.0	63.0	30.0
S13 – Stretch 1	53.5	52.0	20.0
S13 – Stretch 2	79.0	57.5	40.0
S13 – Stretch 3	99.0	74.0	60.0
S14 – Stretch 1	42.0	-1.0	30.0
S14 – Stretch 2	9.0	13.0	30.0
S15 – Stretch 1	83.5	89.0	60.0
S15 – Stretch 2	64.5	111.5	80.0
S15 – Stretch 3	86.0	65.5	60.0
S16 – Stretch 1	39.0	N/A	50.0
S16 – Stretch 2	31.0	52.0	40.0
S16 – Stretch 3	76.0	99.5	80.0
S17 – Stretch 1	54.5	77	60
S17 – Stretch 2	67.0	86.5	80.0
S18 – Stretch 1	142.5	-16.0	150.0
S18 – Stretch 2	60.5	79.5	80
S18 – Stretch 3	64.5	17	80

**Median**	**75.2**	**77.0**	**60.0**
**25th percentile**	**55.9**	**52.0**	**40.0**
**75th percentile**	**104.6**	**99.5**	**80.0**

Position 3 (n=35)

Subject	Onset-Visu	Onset-Auto	MaxAcc
S1 – Stretch 1	204.5	110.0	170.0
S1 – Stretch 2	194.0	179.0	250.0
S1 – Stretch 3	184.0	136.5	210.0
S2 – Stretch 1	233.5	253.0	270.0
S2 – Stretch 2	182.5	195.0	220.0
S2 – Stretch 3	202.0	387.0	330.0
S5 – Stretch 1	119.5	118.5	90.0
S5 – Stretch 2	165.5	170.5	110.0
S6 – Stretch 1	262.5	230.5	250.0
S6 – Stretch 2	206.0	311.5	260.0
S8 – Stretch 1	173.5	172.5	120.0
S8 – Stretch 2	145.5	144.5	100.0
S8 – Stretch 3	179.5	238.0	10.0
S9 – Stretch 1	89.5	94.5	190.0
S9 – Stretch 2	102.5	150.5	190.0
S11 – Stretch 1	382.0	362.0	240.0
S11 – Stretch 2	332.5	388.5	220.0
S12 – Stretch 1	183.5	190.5	120.0
S12 – Stretch 2	170.5	171.0	200.0
S13 – Stretch 1	258.5	258.0	160.0
S13 – Stretch 2	253.0	252.0	90.0
S14 – Stretch 1	203.5	201.5	220.0
S14 – Stretch 2	253.0	263.0	300.0
S14 – Stretch 3	239.0	238.0	290.0
S15 – Stretch 1	147.0	N/A	120.0
S15 – Stretch 2	82.5	-199.0	180.0
S16 – Stretch 1	106.5	-5.0	140.0
S16 – Stretch 2	188.0	-13.5	210.0
S16 – Stretch 3	185.5	N/A	260.0
S17 – Stretch 1	37.0	86.0	140.0
S17 – Stretch 2	85.0	91.0	160.0
S17 – Stretch 3	80.0	83.5	170.0
S18 – Stretch 1	59.0	131.5	150.0
S18 – Stretch 2	189.5	193.5	270.0
S18 – Stretch 3	168.5	175.0	260.0
**Median**	**183.5**	**175.0**	**190.0**
**25th percentile**	**132.5**	**118.5**	**140.0**
**75th percentile**	**205.3**	**238.0**	**250.0**

Subject	Onset-Visu	Onset-Auto	MaxAcc
S1 – Stretch 1	161.0	162.0	150.0
S1 – Stretch 2	277.0	276.0	250.0
S1 – Stretch 3	116.0	122.0	130.0
S2 – Stretch 1	272.5	-195.0	300.0
S2 – Stretch 2	169.5	132.0	280.0
S2 – Stretch 3	161.5	111.5	130.0
S3 – Stretch 1	211.0	232.5	160.0
S3 – Stretch 2	191.0	194.5	100.0
S3 – Stretch 3	334.0	N/A	140.0
S4 – Stretch 1	273.0	279.0	230.0
S4 – Stretch 2	189.0	266.5	100.0
S4 – Stretch 3	426.5	429.5	430.0
S5 – Stretch 1	119.5	101.5	80.0
S5 – Stretch 2	167.0	170.5	140.0
S5 – Stretch 3	140.0	138.0	120.0
S6 – Stretch 1	309.0	171.5	250.0
S6 – Stretch 2	248.0	197.0	110.0
S6 – Stretch 3	272.5	178.5	180.0
S7 – Stretch 1	293.5	294.0	220.0
S7 – Stretch 2	199.5	182.5	270.0
S7 – Stretch 3	334.0	337.5	420.0
S8 – Stretch 1	296.0	286.5	180.0
S8 – Stretch 2	220.0	219.5	120.0
S8 – Stretch 3	321.5	281.5	230.0
S9 – Stretch 1	240.5	243.5	240.0
S9 – Stretch 2	206.5	206.5	190.0
S12 – Stretch 1	188.5	187.5	150.0
S12 – Stretch 2	176.5	184.5	150.0
S12 – Stretch 3	225.0	189.5	180.0
S13 – Stretch 1	225.0	224.5	170.0
S13 – Stretch 2	250.0	250.0	200.0
S13 – Stretch 3	164.5	139.5	90.0
S14 – Stretch 1	279.5	281.0	230.0
S14 – Stretch 2	229.0	131.0	140.0
S14 – Stretch 3	218.5	225.5	190.0
S15 – Stretch 1	270.0	283.0	170.0
S15 – Stretch 2	88.0	81.5	200.0
S15 – Stretch 3	138.0	99.0	170.0
S16 – Stretch 1	356.5	352.5	320.0
S16 – Stretch 2	217.5	223.5	190.0
S16 – Stretch 3	128.5	136.5	60.0
S18 – Stretch 1	174.5	170	170.0
S18 – Stretch 2	237.5	236	250.0
S18 – Stretch 3	346.5	347.5	290.0
**Median**	**222.5**	**197.0**	**180.0**
**25th percentile**	**173.3**	**150.8**	**140.0**
**75th percentile**	**274.0**	**271.3**	**232.5**

Position 4 (n=44)

Table S2: Post-Catch detailed values. Following tables present the delay (in ms) between the timing of the EMG-Onset minus 30ms or maximum a_{mt} values and the catch for the different positions. N/A represents the cases where no EMG-Onset was found. Red cases represent post-catch physiological inconsistencies.

Position 1 (n=37)			
Subject	Onset-Visu	Onset-Auto	MaxAcc
S1 – Stretch 1	102.0	69.5	30.0
S1 – Stretch 2	122.5	93.0	40.0
S2 – Stretch 1	12.0	19.0	50.0
S2 – Stretch 2	28.5	17.5	50.0
S2 – Stretch 3	38.0	38.0	50.0
S3 – Stretch 1	43.0	43.5	80.0
S3 – Stretch 2	39.0	73.0	110.0
S5 – Stretch 1	15.0	22.5	40.0
S5 – Stretch 2	18.0	18.0	80.0
S5 – Stretch 3	13.0	25.0	60.0
S6 – Stretch 1	28.0	40.5	50.0
S6 – Stretch 2	23.0	40.0	50.0
S8 – Stretch 1	17.0	18.0	90.0
S8 – Stretch 2	23.5	22.0	60.0
S9 – Stretch 1	29.5	78.5	50.0
S9 – Stretch 2	17.0	42.0	50.0
S9 – Stretch 3	36.5	33.5	50.0
S10 – Stretch 1	39.0	62.0	60.0
S10 – Stretch 2	35.0	27.5	90.0
S10 – Stretch 3	26.5	53.5	80.0
S11 – Stretch 1	28.0	45.0	140.0
S11 – Stretch 2	-52.5	-54.0	90.0
S12 – Stretch 1	30.5	42.0	20.0
S12 – Stretch 2	41.0	44.0	40.0
S13 – Stretch 1	18.0	21.0	100.0
S13 – Stretch 2	26.0	64.0	100.0
S14 – Stretch 1	31.5	16.5	40.0
S14 – Stretch 2	13.0	12.5	50.0
S14 – Stretch 3	51.0	45.0	50.0
S16 – Stretch 1	48.5	82.5	60.0
S16 – Stretch 2	60.0	60.0	60.0
S17 – Stretch 1	40.5	42.0	40.0
S17 – Stretch 2	35.0	50.0	50.0
S17 – Stretch 3	23.0	82.0	40.0
S18 – Stretch 1	60.5	38.5	40.0
S18 – Stretch 2	61.5	212.0	40.0
S18 – Stretch 3	66.5	108.5	40.0

**Median**	**30.5**	**42.0**	**50.0**
**25th percentile**	**23.5**	**22.5**	**40.0**
**75th percentile**	**41.0**	**62.0**	**80.0**

Position 2 (n=42)

Subject	Onset-Visu	Onset-Auto	MaxAcc
S1 – Stretch 1	94.0	44.5	60.0
S1 – Stretch 2	121.0	293.5	60.0
S2 – Stretch 1	71.0	47.5	60.0
S2 – Stretch 2	77.0	86.0	60.0
S2 – Stretch 3	58.0	53.5	50.0
S3 – Stretch 1	36.0	13.5	80.0
S3 – Stretch 2	3.5	39.5	60.0
S4 – Stretch 1	42.0	31.5	80.0
S4 – Stretch 2	-0.5	15.0	70.0
S4 – Stretch 3	19.0	44.5	70.0
S5 – Stretch 1	65.5	28.0	60.0
S5 – Stretch 2	52.0	418.0	60.0
S5 – Stretch 3	47.0	-117.0	60.0
S6 – Stretch 1	39.0	39.0	50.0
S6 – Stretch 2	30.0	27.5	40.0
S6 – Stretch 3	25.5	8.0	70.0
S8 – Stretch 1	32.0	1.0	130.0
S8 – Stretch 2	33.5	-15.5	90.0
S8 – Stretch 3	52.5	-249.0	40.0
S9 – Stretch 1	43.0	90.5	40.0
S9 – Stretch 2	52.0	55.0	50.0
S9 – Stretch 3	46.0	83.0	50.0
S11 – Stretch 1	-24.5	47.0	80.0
S11 – Stretch 2	-54.0	24.0	40.0
S12 – Stretch 1	57.0	54.0	60.0
S12 – Stretch 2	40.0	37.0	70.0
S13 – Stretch 1	26.5	28.0	60.0
S13 – Stretch 2	11.0	32.5	50.0
S13 – Stretch 3	11.0	36.0	50.0
S14 – Stretch 1	38.0	81.0	50.0
S14 – Stretch 2	71.0	67.0	50.0
S15 – Stretch 1	26.5	21.0	50.0
S15 – Stretch 2	55.5	8.5	40.0
S15 – Stretch 3	34.0	54.5	60.0
S16 – Stretch 1	51.0	N/A	40.0
S16 – Stretch 2	59.0	38.0	50.0
S16 – Stretch 3	34.0	10.5	30.0
S17 – Stretch 1	55.5	33.0	50.0
S17 – Stretch 2	73.0	53.5	60.0
S18 – Stretch 1	17.5	176.0	10.0
S18 – Stretch 2	39.5	20.5	20.0
S18 – Stretch 3	45.5	93	30.0

**Median**	**39.0**	**34.5**	**50.0**
**25th percentile**	**26.5**	**19.1**	**40.0**
**75th percentile**	**52.0**	**54.1**	**60.0**

Position 3 (n=35)

Subject	Onset-Visu	Onset-Auto	MaxAcc
S1 – Stretch 1	105.5	200.0	140.0
S1 – Stretch 2	146.0	161.0	90.0
S1 – Stretch 3	136.0	183.5	110.0
S2 – Stretch 1	86.5	67.0	50.0
S2 – Stretch 2	87.5	75.0	50.0
S2 – Stretch 3	168.0	-17.0	40.0
S5 – Stretch 1	80.5	81.5	110.0
S5 – Stretch 2	104.5	99.5	160.0
S6 – Stretch 1	117.5	149.5	130.0
S6 – Stretch 2	134.0	28.5	80.0
S8 – Stretch 1	-3.5	-2.5	50.0
S8 – Stretch 2	64.5	65.5	110.0
S8 – Stretch 3	30.5	-28.0	200.0
S9 – Stretch 1	90.5	85.5	30.0
S9 – Stretch 2	107.5	59.5	20.0
S11 – Stretch 1	-32.0	-12.0	110.0
S11 – Stretch 2	-22.5	-78.5	90.0
S12 – Stretch 1	86.5	79.5	150.0
S12 – Stretch 2	49.5	49.0	20.0
S13 – Stretch 1	61.5	62.0	160.0
S13 – Stretch 2	-23.0	-22.0	140.0
S14 – Stretch 1	56.5	58.5	40.0
S14 – Stretch 2	77.0	67.0	30.0
S14 – Stretch 3	111.0	112.0	60.0
S15 – Stretch 1	23.0	N/A	50.0
S15 – Stretch 2	117.5	399.0	20.0
S16 – Stretch 1	43.5	155.0	10.0
S16 – Stretch 2	42.0	243.5	20.0
S16 – Stretch 3	154.5	N/A	80.0
S17 – Stretch 1	203.0	154.0	100.0
S17 – Stretch 2	185.0	179.0	110.0
S17 – Stretch 3	110.0	106.5	20.0
S18 – Stretch 1	131.0	58.5	40.0
S18 – Stretch 2	120.5	116.5	40.0
S18 – Stretch 3	121.5	115.0	30.0

**Median**	**90.5**	**79.5**	**60.0**
**25th percentile**	**53.0**	**58.5**	**35.0**
**75th percentile**	**121.0**	**149.5**	**110.0**

**Position 4 (n=44)**			
**Subject**	**Onset-Visu**	**Onset-Auto**	**MaxAcc**
S1 – Stretch 1	129.0	128.0	170.0
S1 – Stretch 2	193.0	194.0	250.0
S1 – Stretch 3	144.0	138.0	160.0
S2 – Stretch 1	137.5	605.0	140.0
S2 – Stretch 2	250.5	288.0	170.0
S2 – Stretch 3	128.5	178.5	190.0
S3 – Stretch 1	9.0	-12.5	90.0
S3 – Stretch 2	-31.0	-34.5	90.0
S3 – Stretch 3	-114.0	N/A	110.0
S4 – Stretch 1	17.0	11.0	90.0
S4 – Stretch 2	151.0	73.5	270.0
S4 – Stretch 3	13.5	10.5	40.0
S5 – Stretch 1	90.5	108.5	160.0
S5 – Stretch 2	83.0	79.5	140.0
S5 – Stretch 3	60.0	62.0	110.0
S6 – Stretch 1	51.0	188.5	140.0
S6 – Stretch 2	42.0	93.0	210.0
S6 – Stretch 3	47.5	141.5	170.0
S7 – Stretch 1	126.5	126.0	230.0
S7 – Stretch 2	100.5	117.5	60.0
S7 – Stretch 3	116.0	112.5	60.0
S8 – Stretch 1	54.0	63.5	200.0
S8 – Stretch 2	80.0	80.5	210.0
S8 – Stretch 3	98.5	138.5	220.0
S9 – Stretch 1	79.5	76.5	110.0
S9 – Stretch 2	53.5	53.5	100.0
S12 – Stretch 1	11.5	12.5	80.0
S12 – Stretch 2	73.5	65.5	130.0
S12 – Stretch 3	15.0	50.5	90.0
S13 – Stretch 1	35.0	35.5	120.0
S13 – Stretch 2	40.0	40.0	120.0
S13 – Stretch 3	25.5	50.5	130.0
S14 – Stretch 1	130.5	129.0	210.0
S14 – Stretch 2	-10.5	99.0	120.0
S14 – Stretch 3	21.5	14.5	80.0
S15 – Stretch 1	20.0	7.0	150.0
S15 – Stretch 2	192.0	198.5	110.0
S15 – Stretch 3	102.0	141.0	100.0
S16 – Stretch 1	63.5	67.5	130.0
S16 – Stretch 2	62.5	56.5	120.0
S16 – Stretch 3	51.5	43.5	150.0
S18 – Stretch 1	55.5	60.0	90.0
S18 – Stretch 2	72.5	74.0	90.0
S18 – Stretch 3	73.5	72.5	160.0

**Median**	**63.0**	**74.0**	**130.0**
**25th percentile**	**32.6**	**50.5**	**97.5**
**75th percentile**	**105.5**	**128.5**	**170.0**

Table S3: T_{vmt} detailed values. Following tables present the T_{vmt} values (in %/s) for the different positions. N/A represents the cases where no EMG-Onset was found or where physiological inconsistencies (pre-T0 or post-catch) were found.

Position 1 (n=37)			
**Subject**	**Onset-Visu**	**Onset-Auto**	**MaxAcc**
S1 – Stretch 1	3.42	12.91	68.70
S1 – Stretch 2	1.69	9.93	71.11
S2 – Stretch 1	34.87	31.45	21.94
S2 – Stretch 2	31.17	35.22	26.61
S2 – Stretch 3	27.42	27.42	27.42
S3 – Stretch 1	34.38	34.38	19.37
S3 – Stretch 2	36.93	32.93	17.94
S5 – Stretch 1	81.59	75.77	64.07
S5 – Stretch 2	67.12	67.12	34.31
S5 – Stretch 3	82.79	81.15	53.80
S6 – Stretch 1	48.13	29.39	29.39
S6 – Stretch 2	62.44	35.83	35.83
S8 – Stretch 1	24.24	24.24	14.70
S8 – Stretch 2	52.04	52.04	30.72
S9 – Stretch 1	62.55	N/A	42.53
S9 – Stretch 2	84.84	52.55	52.55
S9 – Stretch 3	45.38	59.49	45.38
S10 – Stretch 1	21.70	10.55	16.09
S10 – Stretch 2	29.62	29.62	11.25
S10 – Stretch 3	24.62	18.91	12.39
S11 – Stretch 1	68.42	78.67	34.94
S11 – Stretch 2	N/A	N/A	29.30
S12 – Stretch 1	11.71	6.41	31.11
S12 – Stretch 2	11.79	11.79	19.55
S13 – Stretch 1	66.62	66.62	22.60
S13 – Stretch 2	69.99	63.06	39.19
S14 – Stretch 1	70.55	84.47	70.55
S14 – Stretch 2	138.18	138.18	88.86
S14 – Stretch 3	47.06	62.29	62.29
S16 – Stretch 1	38.25	8.17	38.25
S16 – Stretch 2	27.67	27.67	39.40
S17 – Stretch 1	29.83	29.83	43.45
S17 – Stretch 2	63.23	26.38	44.15
S17 – Stretch 3	67.97	1.28	52.49
S18 – Stretch 1	19.96	39.74	54.10
S18 – Stretch 2	26.32	N/A	62.07
S18 – Stretch 3	10.63	N/A	73.17
**Median**	**37.59**	**32.93**	**38.25**
**25th percentile**	**25.90**	**24.24**	**26.61**
**75th percentile**	**66.75**	**62.29**	**53.80**

Position 2 (n=42)

Subject	Onset-Visu	Onset-Auto	MaxAcc
S1 – Stretch 1	N/A	30.16	19.87
S1 – Stretch 2	N/A	N/A	22.43
S2 – Stretch 1	14.10	26.18	26.18
S2 – Stretch 2	8.42	5.46	29.59
S2 – Stretch 3	10.42	19.30	30.40
S3 – Stretch 1	30.98	37.14	17.62
S3 – Stretch 2	34.76	22.99	17.45
S4 – Stretch 1	29.28	31.51	16.00
S4 – Stretch 2	N/A	33.32	22.57
S4 – Stretch 3	38.76	33.04	20.11
S5 – Stretch 1	18.78	38.05	25.29
S5 – Stretch 2	28.47	N/A	28.47
S5 – Stretch 3	23.81	N/A	23.81
S6 – Stretch 1	27.71	27.71	27.71
S6 – Stretch 2	26.38	26.38	26.38
S6 – Stretch 3	35.19	36.49	15.74
S8 – Stretch 1	18.79	36.01	4.64
S8 – Stretch 2	14.30	N/A	8.10
S8 – Stretch 3	7.68	N/A	13.45
S9 – Stretch 1	22.06	2.57	31.80
S9 – Stretch 2	18.91	18.91	25.70
S9 – Stretch 3	20.50	4.25	29.12
S11 – Stretch 1	N/A	49.46	33.09
S11 – Stretch 2	N/A	54.11	45.90
S12 – Stretch 1	17.10	24.30	24.30
S12 – Stretch 2	27.09	27.09	17.87
S13 – Stretch 1	41.52	41.52	23.36
S13 – Stretch 2	80.63	59.71	47.12
S13 – Stretch 3	65.00	37.50	37.50
S14 – Stretch 1	39.14	N/A	39.14
S14 – Stretch 2	9.52	9.52	40.67
S15 – Stretch 1	40.26	47.36	31.00
S15 – Stretch 2	18.05	44.65	31.98
S15 – Stretch 3	54.50	36.07	36.07
S16 – Stretch 1	11.31	N/A	25.33
S16 – Stretch 2	11.71	32.15	32.15
S16 – Stretch 3	25.85	45.14	35.46
S17 – Stretch 1	20.78	37.09	29.23
S17 – Stretch 2	12.48	28.21	28.21
S18 – Stretch 1	10.55	N/A	27.17
S18 – Stretch 2	9.70	25.41	38.94
S18 – Stretch 3	16.71	2.35	41.73
**Median**	**20.78**	**31.83**	**27.44**
**25th percentile**	**14.10**	**24.58**	**22.46**
**75th percentile**	**30.98**	**37.41**	**32.11**

Position 3 (n=35)

Subject	Onset-Visu	Onset-Auto	MaxAcc
S1 – Stretch 1	62.48	10.07	44.85
S1 – Stretch 2	15.32	15.50	43.21
S1 – Stretch 3	13.98	5.72	31.06
S2 – Stretch 1	17.62	21.96	36.47
S2 – Stretch 2	23.22	28.78	42.46
S2 – Stretch 3	3.35	N/A	40.56
S5 – Stretch 1	28.54	28.54	16.77
S5 – Stretch 2	17.73	17.73	13.47
S6 – Stretch 1	28.51	19.69	28.51
S6 – Stretch 2	29.79	45.70	40.73
S8 – Stretch 1	N/A	N/A	19.04
S8 – Stretch 2	18.46	18.46	11.50
S8 – Stretch 3	21.24	N/A	4.72
S9 – Stretch 1	9.55	11.68	36.12
S9 – Stretch 2	14.31	14.89	39.09
S11 – Stretch 1	N/A	N/A	30.03
S11 – Stretch 2	N/A	N/A	29.22
S12 – Stretch 1	17.47	16.56	14.32
S12 – Stretch 2	17.73	17.73	42.26
S13 – Stretch 1	49.28	49.28	17.96
S13 – Stretch 2	N/A	N/A	15.46
S14 – Stretch 1	14.61	14.61	27.39
S14 – Stretch 2	11.35	12.89	37.82
S14 – Stretch 3	18.66	18.66	42.41
S15 – Stretch 1	66.63	N/A	48.32
S15 – Stretch 2	10.33	N/A	54.51
S16 – Stretch 1	25.49	N/A	38.26
S16 – Stretch 2	29.37	N/A	40.82
S16 – Stretch 3	10.08	N/A	30.51
S17 – Stretch 1	4.76	19.83	56.47
S17 – Stretch 2	14.70	14.70	56.69
S17 – Stretch 3	8.94	8.94	59.32
S18 – Stretch 1	5.91	18.29	44.39
S18 – Stretch 2	14.62	14.62	46.56
S18 – Stretch 3	7.69	11.93	42.25

**Median**	**17.47**	**17.15**	**38.26**
**25th percentile**	**10.84**	**14.18**	**27.95**
**75th percentile**	**24.35**	**19.73**	**42.84**

Position 4 (n=44)			
**Subject**	**Onset-Visu**	**Onset-Auto**	**MaxAcc**
S1 – Stretch 1	40.91	40.91	40.91
S1 – Stretch 2	61.36	61.36	44.59
S1 – Stretch 3	26.50	26.50	46.26
S2 – Stretch 1	27.41	N/A	39.37
S2 – Stretch 2	6.04	2.55	22.91
S2 – Stretch 3	24.69	10.76	18.90
S3 – Stretch 1	25.58	N/A	17.14
S3 – Stretch 2	N/A	N/A	13.26
S3 – Stretch 3	N/A	N/A	14.97
S4 – Stretch 1	28.07	28.48	21.36
S4 – Stretch 2	24.69	16.75	11.09
S4 – Stretch 3	19.65	19.65	21.76
S5 – Stretch 1	27.04	20.64	16.77
S5 – Stretch 2	27.54	27.54	20.27
S5 – Stretch 3	26.29	26.29	23.72
S6 – Stretch 1	25.42	15.61	23.37
S6 – Stretch 2	28.35	18.68	13.69
S6 – Stretch 3	15.48	14.01	20.47
S7 – Stretch 1	19.58	19.58	16.04
S7 – Stretch 2	23.90	21.25	20.08
S7 – Stretch 3	25.57	25.10	20.49
S8 – Stretch 1	17.12	16.58	12.16
S8 – Stretch 2	18.58	18.58	10.67
S8 – Stretch 3	16.71	16.44	12.57
S9 – Stretch 1	4.94	4.94	5.71
S9 – Stretch 2	11.58	11.58	9.85
S12 – Stretch 1	33.78	33.78	26.46
S12 – Stretch 2	52.82	58.39	37.00
S12 – Stretch 3	44.30	32.87	32.87
S13 – Stretch 1	27.94	26.26	16.88
S13 – Stretch 2	36.16	36.16	23.26
S13 – Stretch 3	41.16	38.21	18.71
S14 – Stretch 1	22.07	22.07	16.73
S14 – Stretch 2	N/A	13.01	16.57
S14 – Stretch 3	31.15	33.52	25.82
S15 – Stretch 1	34.88	35.14	17.40
S15 – Stretch 2	1.96	1.34	12.80
S15 – Stretch 3	13.89	7.91	24.90
S16 – Stretch 1	25.67	24.04	19.80
S16 – Stretch 2	31.32	31.32	26.47
S16 – Stretch 3	32.58	34.56	16.72
S18 – Stretch 1	25.27	22.07	25.27
S18 – Stretch 2	17.82	17.82	25.59
S18 – Stretch 3	24.12	24.12	25.13

**Median**	**25.58**	**22.07**	**20.18**
**25th percentile**	**19.58**	**16.54**	**16.44**
**75th percentile**	**31.15**	**31.71**	**25.17**

Table S4: Number of T_{vmt} exceedances per gait cycle – detailed values. Following tables present the number of T_{vmt} exceedances per gait cycle (in /gait cycle) for the different positions. N/A represents the cases where no EMG-Onset was found or where physiological inconsistencies (pre-T0 or post-catch) were found.

Position 1 (n=37)			
**Subject**	**Onset-Visu**	**Onset-Auto**	**MaxAcc**
S1 – Stretch 1	2.75	2.00	0.00
S1 – Stretch 2	2.75	2.75	0.00
S2 – Stretch 1	0.00	0.00	0.80
S2 – Stretch 2	0.00	0.00	0.20
S2 – Stretch 3	0.20	0.20	0.20
S3 – Stretch 1	0.00	0.00	1.00
S3 – Stretch 2	0.00	0.33	1.00
S5 – Stretch 1	0.00	0.00	0.00
S5 – Stretch 2	0.00	0.00	0.00
S5 – Stretch 3	0.00	0.00	0.00
S6 – Stretch 1	0.00	0.00	0.00
S6 – Stretch 2	0.00	0.00	0.00
S8 – Stretch 1	1.00	1.00	1.00
S8 – Stretch 2	0.00	0.00	1.00
S9 – Stretch 1	0.00	N/A	0.00
S9 – Stretch 2	0.00	0.00	0.00
S9 – Stretch 3	0.00	0.00	0.00
S10 – Stretch 1	0.25	1.50	1.00
S10 – Stretch 2	0.00	0.00	1.50
S10 – Stretch 3	0.00	0.75	1.50
S11 – Stretch 1	0.00	0.00	0.00
S11 – Stretch 2	N/A	N/A	0.00
S12 – Stretch 1	1.25	2.50	1.00
S12 – Stretch 2	1.25	1.25	1.25
S13 – Stretch 1	0.00	0.00	0.00
S13 – Stretch 2	0.00	0.00	0.00
S14 – Stretch 1	0.00	0.00	0.00
S14 – Stretch 2	0.00	0.00	0.00
S14 – Stretch 3	0.00	0.00	0.00
S16 – Stretch 1	0.00	1.25	0.00
S16 – Stretch 2	0.00	0.00	0.00
S17 – Stretch 1	0.25	0.25	0.00
S17 – Stretch 2	0.00	0.25	0.00
S17 – Stretch 3	0.00	3.00	0.00
S18 – Stretch 1	1.00	1.00	0.00
S18 – Stretch 2	1.00	N/A	0.00
S18 – Stretch 3	1.00	N/A	0.00
**Median**	**0.00**	**0.00**	**0.00**
**25th percentile**	**0.00**	**0.00**	**0.00**
**75th percentile**	**0.25**	**1.00**	**0.80**

Position 2 (n=42)

**Subject**	**Onset-Visu**	**Onset-Auto**	**MaxAcc**
S1 – Stretch 1	N/A	1.75	1.75
S1 – Stretch 2	N/A	N/A	1.75
S2 – Stretch 1	0.80	0.00	0.00
S2 – Stretch 2	0.00	0.00	0.00
S2 – Stretch 3	0.00	0.00	0.00
S3 – Stretch 1	0.00	0.00	0.67
S3 – Stretch 2	0.00	0.00	0.67
S4 – Stretch 1	0.00	0.00	1.00
S4 – Stretch 2	N/A	0.00	0.20
S4 – Stretch 3	0.00	0.00	0.60
S5 – Stretch 1	0.60	0.00	0.20
S5 – Stretch 2	0.20	N/A	0.20
S5 – Stretch 3	0.20	N/A	0.20
S6 – Stretch 1	0.00	0.00	0.00
S6 – Stretch 2	0.00	0.00	0.00
S6 – Stretch 3	0.00	0.00	1.00
S8 – Stretch 1	1.00	0.75	1.00
S8 – Stretch 2	1.00	N/A	1.00
S8 – Stretch 3	1.00	N/A	0.75
S9 – Stretch 1	0.67	6.00	0.00
S9 – Stretch 2	0.83	0.83	0.33
S9 – Stretch 3	0.67	6.00	0.17
S11 – Stretch 1	N/A	0.00	0.00
S11 – Stretch 2	N/A	0.00	0.00
S12 – Stretch 1	4.00	4.00	4.00
S12 – Stretch 2	4.00	4.00	4.00
S13 – Stretch 1	0.75	0.75	1.00
S13 – Stretch 2	0.00	0.25	0.50
S13 – Stretch 3	0.25	1.00	1.00
S14 – Stretch 1	0.00	N/A	0.00
S14 – Stretch 2	1.50	1.50	0.00
S15 – Stretch 1	0.00	0.00	0.25
S15 – Stretch 2	1.00	0.00	0.25
S15 – Stretch 3	0.00	0.00	0.00
S16 – Stretch 1	2.00	N/A	0.25
S16 – Stretch 2	1.75	0.00	0.00
S16 – Stretch 3	0.25	0.00	0.00
S17 – Stretch 1	2.50	1.75	1.75
S17 – Stretch 2	2.50	1.75	1.75
S18 – Stretch 1	1.00	N/A	1.00
S18 – Stretch 2	0.75	1.00	0.00
S18 – Stretch 3	0.75	0.50	0.00
**Median**	0.67	0.00	0.25
**25th percentile**	0.00	0.00	0.00
**75th percentile**	1.00	1.00	1.00

Position 3 (n=35)

Subject	Onset-Visu	Onset-Auto	MaxAcc
S1 – Stretch 1	0.00	1.00	1.00
S1 – Stretch 2	1.00	1.00	1.00
S1 – Stretch 3	1.00	1.00	2.00
S2 – Stretch 1	0.20	0.80	1.00
S2 – Stretch 2	0.80	1.00	1.00
S2 – Stretch 3	0.60	N/A	1.00
S5 – Stretch 1	0.40	0.40	1.20
S5 – Stretch 2	1.00	1.00	1.20
S6 – Stretch 1	1.00	0.33	1.00
S6 – Stretch 2	1.00	0.33	1.00
S8 – Stretch 1	N/A	N/A	1.50
S8 – Stretch 2	1.50	1.50	1.50
S8 – Stretch 3	1.25	N/A	2.75
S9 – Stretch 1	3.17	3.17	0.00
S9 – Stretch 2	2.67	2.67	0.00
S11 – Stretch 1	N/A	N/A	1.00
S11 – Stretch 2	N/A	N/A	1.00
S12 – Stretch 1	0.25	0.00	0.00
S12 – Stretch 2	0.25	0.25	1.50
S13 – Stretch 1	0.75	0.75	0.00
S13 – Stretch 2	N/A	N/A	0.00
S14 – Stretch 1	1.00	1.00	1.00
S14 – Stretch 2	1.00	1.00	1.00
S14 – Stretch 3	1.00	1.00	1.00
S15 – Stretch 1	1.00	N/A	0.00
S15 – Stretch 2	1.00	N/A	0.00
S16 – Stretch 1	0.00	N/A	0.00
S16 – Stretch 2	0.00	N/A	0.00
S16 – Stretch 3	0.00	N/A	0.00
S17 – Stretch 1	0.50	0.75	1.00
S17 – Stretch 2	0.50	0.50	1.00
S17 – Stretch 3	0.00	0.00	1.00
S18 – Stretch 1	1.00	0.25	1.00
S18 – Stretch 2	0.50	0.50	1.00
S18 – Stretch 3	1.00	0.75	1.00

**Median**	**1.00**	**0.78**	**1.00**
**25th percentile**	**0.33**	**0.38**	**0.00**
**75th percentile**	**1.00**	**1.00**	**1.00**

Subject	Onset-Visu	Onset-Auto	MaxAcc
S1 – Stretch 1	0.25	0.25	0.25
S1 – Stretch 2	0.00	0.00	0.25
S1 – Stretch 3	1.00	1.00	0.25
S2 – Stretch 1	0.00	N/A	0.00
S2 – Stretch 2	2.00	3.00	0.20
S2 – Stretch 3	0.00	2.00	0.80
S3 – Stretch 1	0.33	N/A	1.00
S3 – Stretch 2	N/A	N/A	0.67
S3 – Stretch 3	N/A	N/A	0.67
S4 – Stretch 1	0.00	0.00	0.00
S4 – Stretch 2	0.00	0.00	0.00
S4 – Stretch 3	0.00	0.00	0.00
S5 – Stretch 1	0.00	0.00	0.00
S5 – Stretch 2	0.00	0.00	0.00
S5 – Stretch 3	0.00	0.00	0.00
S6 – Stretch 1	0.00	1.33	0.00
S6 – Stretch 2	0.00	0.67	1.33
S6 – Stretch 3	1.33	1.33	0.33
S7 – Stretch 1	0.00	0.00	0.00
S7 – Stretch 2	0.00	0.00	0.00
S7 – Stretch 3	0.00	0.00	0.00
S8 – Stretch 1	0.75	0.75	1.75
S8 – Stretch 2	0.50	0.50	2.50
S8 – Stretch 3	0.75	0.75	2.00
S9 – Stretch 1	3.00	3.00	2.00
S9 – Stretch 2	0.17	0.17	0.33
S12 – Stretch 1	0.00	0.00	0.75
S12 – Stretch 2	0.00	0.00	0.00
S12 – Stretch 3	0.00	0.00	0.00
S13 – Stretch 1	0.75	0.75	1.00
S13 – Stretch 2	0.50	0.50	1.00
S13 – Stretch 3	0.50	0.50	1.00
S14 – Stretch 1	0.00	0.00	1.00
S14 – Stretch 2	N/A	1.50	1.00
S14 – Stretch 3	0.00	0.00	0.00
S15 – Stretch 1	0.75	0.75	1.25
S15 – Stretch 2	1.00	1.00	1.75
S15 – Stretch 3	1.50	1.00	1.00
S16 – Stretch 1	0.00	0.00	0.00
S16 – Stretch 2	0.00	0.00	0.00
S16 – Stretch 3	0.00	0.00	0.25
S18 – Stretch 1	0.25	1.00	0.25
S18 – Stretch 2	1.25	1.25	0.25
S18 – Stretch 3	0.50	0.50	0.25

**Median**	**0.00**	**0.38**	**0.25**
**25th percentile**	**0.00**	**0.00**	**0.00**
**75th percentile**	**0.75**	**1.00**	**1.00**

Position 4 (n=44)

Table S5: Proportion of the gait trial time where v_{mt} is superior to T_{vmt} – detailed values. Following tables present the proportion of the gait trial time where v_{mt} is superior to T_{vmt} values (in %) for the different positions. N/A represents the cases where no EMG-Onset was found or where physiological inconsistencies (pre-T0 or post-catch) were found.

Position 1 (n=37)			
**Subject**	**Onset-Visu**	**Onset-Auto**	**MaxAcc**
S1 – Stretch 1	36.39	14.76	0.00
S1 – Stretch 2	39.69	24.17	0.00
S2 – Stretch 1	0.00	0.00	3.73
S2 – Stretch 2	0.00	0.00	0.66
S2 – Stretch 3	0.66	0.66	0.66
S3 – Stretch 1	0.00	0.00	5.88
S3 – Stretch 2	0.00	0.31	6.81
S5 – Stretch 1	0.00	0.00	0.00
S5 – Stretch 2	0.00	0.00	0.00
S5 – Stretch 3	0.00	0.00	0.00
S6 – Stretch 1	0.00	0.00	0.00
S6 – Stretch 2	0.00	0.00	0.00
S8 – Stretch 1	5.22	5.22	7.21
S8 – Stretch 2	0.00	0.00	5.47
S9 – Stretch 1	0.00	N/A	0.00
S9 – Stretch 2	0.00	0.00	0.00
S9 – Stretch 3	0.00	0.00	0.00
S10 – Stretch 1	0.48	9.26	2.61
S10 – Stretch 2	0.00	0.00	8.79
S10 – Stretch 3	0.00	0.95	8.31
S11 – Stretch 1	0.00	0.00	0.00
S11 – Stretch 2	N/A	N/A	0.00
S12 – Stretch 1	10.51	15.40	3.67
S12 – Stretch 2	10.27	10.27	8.07
S13 – Stretch 1	0.00	0.00	0.00
S13 – Stretch 2	0.00	0.00	0.00
S14 – Stretch 1	0.00	0.00	0.00
S14 – Stretch 2	0.00	0.00	0.00
S14 – Stretch 3	0.00	0.00	0.00
S16 – Stretch 1	0.00	10.23	0.00
S16 – Stretch 2	0.00	0.00	0.00
S17 – Stretch 1	0.49	0.49	0.00
S17 – Stretch 2	0.00	0.74	0.00
S17 – Stretch 3	0.00	34.31	0.00
S18 – Stretch 1	7.69	2.10	0.00
S18 – Stretch 2	6.06	N/A	0.00
S18 – Stretch 3	10.49	N/A	0.00
**Median**	**0.00**	**0.00**	**0.00**
**25th percentile**	**0.00**	**0.00**	**0.00**
**75th percentile**	**0.53**	**2.10**	**2.61**

Position 2 (n=42)			
**Subject**	**Onset-Visu**	**Onset-Auto**	**MaxAcc**
S1 – Stretch 1	N/A	5.60	8.65
S1 – Stretch 2	N/A	N/A	8.14
S2 – Stretch 1	3.07	0.00	0.00
S2 – Stretch 2	N/A	N/A	0.00
S2 – Stretch 3	N/A	N/A	0.00
S3 – Stretch 1	0.00	0.00	1.24
S3 – Stretch 2	0.00	0.00	1.55
S4 – Stretch 1	0.00	0.00	2.60
S4 – Stretch 2	N/A	0.00	0.45
S4 – Stretch 3	0.00	0.00	1.02
S5 – Stretch 1	1.16	0.00	0.32
S5 – Stretch 2	0.21	N/A	0.21
S5 – Stretch 3	0.42	N/A	0.42
S6 – Stretch 1	0.00	0.00	0.00
S6 – Stretch 2	0.00	0.00	0.00
S6 – Stretch 3	0.00	0.00	2.52
S8 – Stretch 1	6.72	1.99	3.48
S8 – Stretch 2	7.71	N/A	5.22
S8 – Stretch 3	5.47	N/A	8.96
S9 – Stretch 1	0.73	28.09	0.00
S9 – Stretch 2	1.36	1.36	0.31
S9 – Stretch 3	0.84	19.81	0.10
S11 – Stretch 1	N/A	0.00	0.00
S11 – Stretch 2	N/A	0.00	0.00
S12 – Stretch 1	15.89	12.22	12.22
S12 – Stretch 2	11.00	11.00	15.16
S13 – Stretch 1	1.81	1.81	5.17
S13 – Stretch 2	0.00	0.26	1.29
S13 – Stretch 3	0.26	3.36	3.36
S14 – Stretch 1	0.00	N/A	0.00
S14 – Stretch 2	8.42	8.42	0.00
S15 – Stretch 1	0.00	0.00	0.63
S15 – Stretch 2	7.56	0.00	0.42
S15 – Stretch 3	0.00	0.00	0.00
S16 – Stretch 1	9.53	N/A	0.23
S16 – Stretch 2	9.07	0.00	0.00
S16 – Stretch 3	0.23	0.00	0.00
S17 – Stretch 1	14.46	7.11	10.29
S17 – Stretch 2	18.63	10.78	10.78
S18 – Stretch 1	11.66	N/A	5.13
S18 – Stretch 2	12.12	5.83	0.00
S18 – Stretch 3	7.69	0.70	0.00
**Median**	**1.16**	**0.00**	**0.42**
**25th percentile**	**0.00**	**0.00**	**0.00**
**75th percentile**	**8.06**	**5.66**	**3.45**

Position 3 (n=35)			
Subject	Onset-Visu	Onset-Auto	MaxAcc
S1 – Stretch 1	0.00	4.83	4.07
S1 – Stretch 2	3.82	3.82	4.33
S1 – Stretch 3	4.07	5.60	8.91
S2 – Stretch 1	2.19	8.77	5.04
S2 – Stretch 2	8.11	7.68	2.85
S2 – Stretch 3	12.06	N/A	3.51
S5 – Stretch 1	0.95	0.95	6.12
S5 – Stretch 2	5.49	5.49	6.55
S6 – Stretch 1	14.78	6.92	14.78
S6 – Stretch 2	14.47	1.26	5.66
S8 – Stretch 1	N/A	N/A	11.94
S8 – Stretch 2	11.94	11.94	14.93
S8 – Stretch 3	10.70	N/A	16.17
S9 – Stretch 1	16.04	12.89	0.00
S9 – Stretch 2	9.64	8.49	0.00
S11 – Stretch 1	N/A	N/A	16.18
S11 – Stretch 2	N/A	N/A	16.38
S12 – Stretch 1	6.36	0.00	0.00
S12 – Stretch 2	6.36	6.36	14.67
S13 – Stretch 1	10.34	10.34	0.00
S13 – Stretch 2	N/A	N/A	0.00
S14 – Stretch 1	17.84	17.84	12.63
S14 – Stretch 2	19.04	18.44	9.22
S14 – Stretch 3	16.03	16.03	7.82
S15 – Stretch 1	4.83	N/A	0.00
S15 – Stretch 2	23.95	N/A	0.00
S16 – Stretch 1	0.00	N/A	0.00
S16 – Stretch 2	0.00	N/A	0.00
S16 – Stretch 3	0.00	N/A	0.00
S17 – Stretch 1	0.98	10.78	6.13
S17 – Stretch 2	7.84	7.84	5.88
S17 – Stretch 3	0.00	0.00	4.90
S18 – Stretch 1	10.72	3.26	5.83
S18 – Stretch 2	4.20	4.20	5.83
S18 – Stretch 3	9.09	5.83	6.99
**Median**	**7.84**	**6.64**	**5.83**
**25th percentile**	**3.00**	**4.10**	**0.00**
**75th percentile**	**12.00**	**10.45**	**9.06**

Position 4 (n=44)			
Subject	Onset-Visu	Onset-Auto	MaxAcc
S1 – Stretch 1	0.00	0.00	0.00
S1 – Stretch 2	0.00	0.00	0.00
S1 – Stretch 3	3.31	3.31	0.00
S2 – Stretch 1	0.00	N/A	0.00
S2 – Stretch 2	32.89	45.61	0.22
S2 – Stretch 3	0.00	23.68	5.04
S3 – Stretch 1	0.93	N/A	7.74
S3 – Stretch 2	N/A	N/A	6.19
S3 – Stretch 3	N/A	N/A	5.88
S4 – Stretch 1	0.00	0.00	0.00
S4 – Stretch 2	0.00	0.00	0.00
S4 – Stretch 3	0.00	0.00	0.00
S5 – Stretch 1	0.00	0.00	0.00
S5 – Stretch 2	0.00	0.00	0.00
S5 – Stretch 3	0.00	0.00	0.00
S6 – Stretch 1	0.00	14.47	0.00
S6 – Stretch 2	0.00	5.66	17.92
S6 – Stretch 3	14.78	16.67	1.57
S7 – Stretch 1	0.00	0.00	0.00
S7 – Stretch 2	0.00	0.00	0.00
S7 – Stretch 3	0.00	0.00	0.00
S8 – Stretch 1	1.24	2.24	8.96
S8 – Stretch 2	1.00	1.00	18.91
S8 – Stretch 3	2.24	2.24	9.45
S9 – Stretch 1	10.48	10.48	5.45
S9 – Stretch 2	0.21	0.21	0.31
S12 – Stretch 1	0.00	0.00	0.73
S12 – Stretch 2	0.00	0.00	0.00
S12 – Stretch 3	0.00	0.00	0.00
S13 – Stretch 1	4.65	4.91	10.34
S13 – Stretch 2	1.81	1.81	7.75
S13 – Stretch 3	0.78	1.29	9.30
S14 – Stretch 1	0.00	0.00	5.81
S14 – Stretch 2	N/A	13.63	6.21
S14 – Stretch 3	0.00	0.00	0.00
S15 – Stretch 1	1.89	1.68	12.18
S15 – Stretch 2	56.72	57.35	18.91
S15 – Stretch 3	13.24	31.30	7.14
S16 – Stretch 1	0.00	0.00	0.00
S16 – Stretch 2	0.00	0.00	0.00
S16 – Stretch 3	0.00	0.00	0.70
S18 – Stretch 1	0.23	2.80	0.23
S18 – Stretch 2	7.46	7.46	0.23
S18 – Stretch 3	0.93	0.93	0.23
**Median**	**0.00**	**0.57**	**0.23**
**25th percentile**	**0.00**	**0.00**	**0.00**
**75th percentile**	**1.81**	**5.10**	**6.45**
